# Chronic Smoking Impairs Glymphatic Transport and Cognitive Function in Adolescent Mice Through Cardiac, Vascular, and Perivascular Aquaporin‐4 Mechanisms

**DOI:** 10.1002/cns.71040

**Published:** 2026-08-03

**Authors:** Chuanxiang Lv, Chengyu Xia, Zean Li, Tao Liu, Zhuang Sha, Shiying Dong, Yu Qian, Mingqi Liu, Kang Zhang, Xinran Zhang, Rongcai Jiang, Li Bie

**Affiliations:** ^1^ Department of Neurosurgery The First Hospital of Jilin University Changchun China; ^2^ Department of Neurosurgery The First Affiliated Hospital of Guangzhou Medical University Guangzhou China; ^3^ Department of Neurosurgery Tianjin Medical University General Hospital Tianjin China; ^4^ Department of Critical Care Medicine Tianjin Medical University General Hospital Tianjin China

**Keywords:** arterial pulsatility, chronic smoking exposure, cognitive impairment, glymphatic system, perivascular AQP4

## Abstract

**Aims:**

Smoking significantly impairs cognitive function and is a major risk factor for dementia, particularly when initiated during adolescence, a critical period for brain development. The glymphatic system, which is thought to participate in metabolic waste clearance, has been implicated in maintaining cognitive health. This study investigates the effects of chronic smoking on glymphatic transport and its underlying mechanisms.

**Methods:**

Glymphatic transport was assessed using immunofluorescence and two‐photon microscopy, while phosphorylated tau accumulation in the dentate gyrus (DG) was examined via immunohistochemical staining. Synaptogenesis and neurogenesis in the hippocampal DG were analyzed using synaptophysin, PSD‐95, doublecortin, and standard histological techniques. Cognitive function was measured through the Morris water maze (MWM) test and novel object recognition (NOR) tests, with cardiac function assessed by echocardiography.

**Results:**

The findings indicate that chronic smoking leads to a duration‐dependent disruption of glymphatic transport, resulting in the accumulation of phosphorylated tau in the hippocampal DG, reduced synaptogenesis and neurogenesis, and subsequent cognitive decline.

**Conclusion:**

This glymphatic dysfunction may be associated with impaired cardiac ejection, diminished arterial pulsatility, and loss of perivascular aquaporin‐4 (AQP4), collectively contributing to smoking‐related cognitive impairment.

## Graphical abstract

Chronic smoking during adolescence impairs glymphatic transport, leading to cognitive decline and aberrant accumulation of phosphorylated tau in the hippocampal DG region. Smoking‐induced glymphatic dysfunction compromises synaptogenesis and neurogenesis in the hippocampal DG region. Compromised cardiac ejection function, reduced arterial pulsatility, and loss of perivascular AQP4 collectively contribute to smoking‐induced glymphatic impairment.

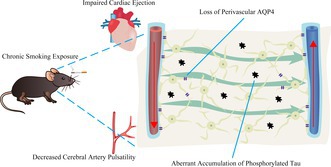

## 1 Introduction

Cigarette smoking remains one of the most pressing public health challenges worldwide. As a significant risk factor endangering human health and life, smoking not only leads to lung diseases but also triggers a variety of other illnesses, resulting in substantial mortality [1]. Research has identified over 4500 toxic chemicals in cigarette smoke, many of which have detrimental effects on human health. Beyond its well‐documented impact on the lungs and cardiovascular system, smoking is also strongly linked to neurological disorders [2].

Recent research has proposed a brain‐wide fluid transport and waste clearance pathway network known as the glymphatic system. This proposed system is hypothesized to utilize perivascular channels formed by astrocytic end‐feet surrounding arteries to facilitate fluid exchange between cerebrospinal fluid (CSF) and interstitial fluid (ISF), thereby clearing metabolic waste and interstitial solutes from brain tissue to maintain central nervous system (CNS) homeostasis [3, 4]. The predominant model of glymphatic transport posits that glymphatic influx into and through the brain is mediated, at least in part, by a perivascular pumping mechanism. This mechanism is driven by the pulsatility of the arterial walls, which are elicited by the cardiac cycle [5, 6]. Within the brain, bulk flow drainage via the glymphatic system is thought to be propelled by cerebral vascular pulsatility and relies on astroglial water channels along paravascular CSF pathways [7]. Within the glymphatic system, a convective influx of CSF is counterbalanced by perivenous efflux of ISF, facilitating the clearance of the neuropil from toxic proteinaceous metabolites such as amyloid‐beta (Aβ) and hyperphosphorylated tau [4, 8, 9].

Glymphatic dysfunction, characterized by impaired clearance of interstitial solutes, is a key feature of natural brain aging and is implicated in various CNS disorders, including Alzheimer's disease (AD), traumatic brain injury (TBI), and both ischemic and hemorrhagic strokes [10]. Glymphatic dysfunction occurs when there is a reduction in cerebral vascular pulsatility or disruption of aquaporin‐4 (AQP4) polarization, leading to decreased clearance of toxic metabolic byproducts and subsequent exacerbation of cognitive decline [7, 8, 9, 10]. In this study, we provide preliminary insights into the relationship between smoking and glymphatic system impairment, elucidating the mechanisms through which cigarette exposure contributes to cognitive dysfunction.

## 2 Materials and Methods

### 2.1 Animals

Adolescent male C57BL/6 mice (5 weeks old, 18–22 g; HFK Bioscience Corporation) were used in accordance with previous studies [11]. Anesthesia was induced with 2%–4% isoflurane and maintained at 1.5% in oxygen‐enriched air (20% O_2_/80% air) under spontaneous ventilation during all invasive procedures.

### 2.2 Cigarette Smoke Exposure and Experiment Design

**FIGURE 1:**
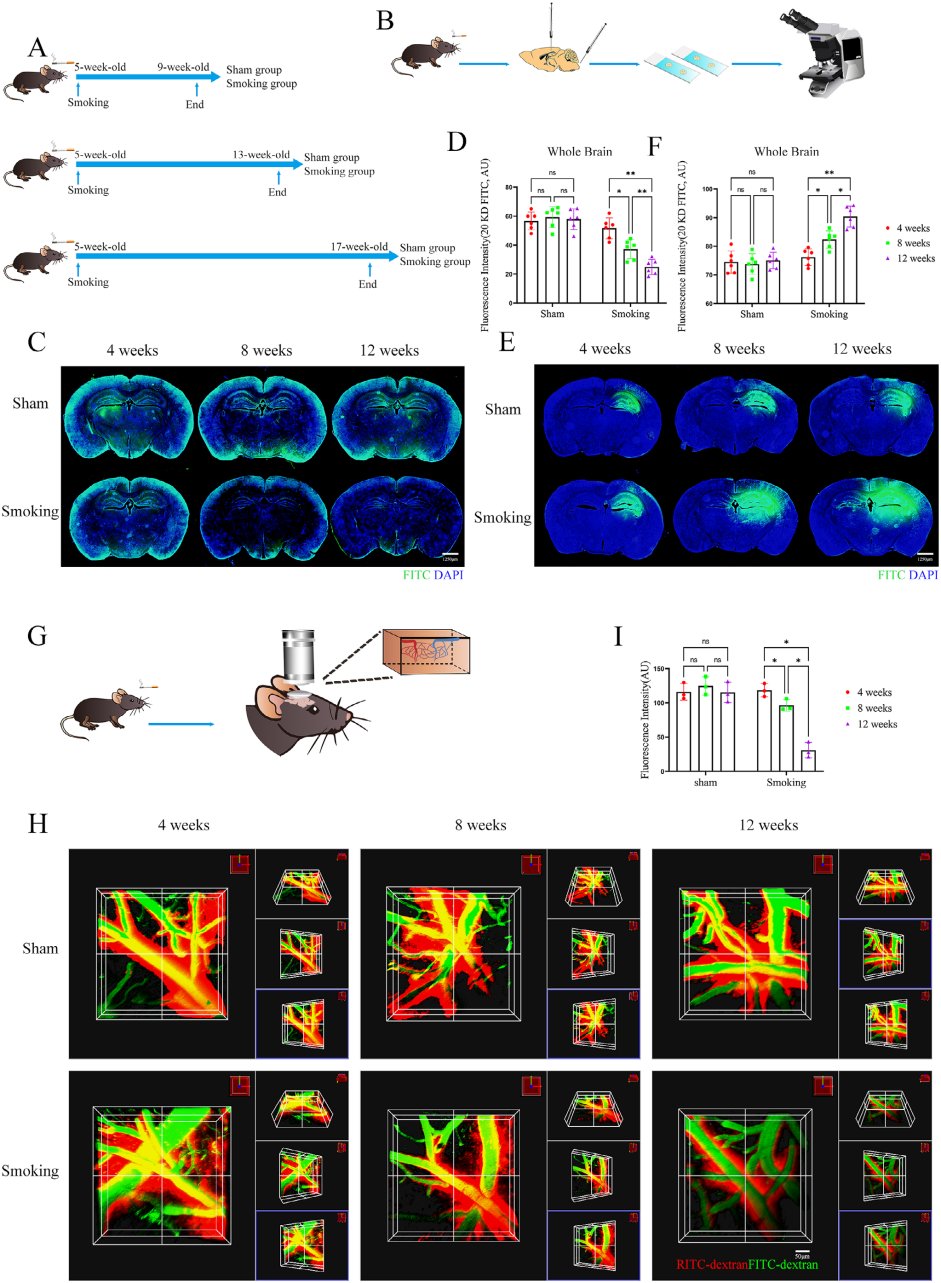
Duration‐dependent impairment of glymphatic transport due to chronic smoking in adolescence. (A) Experimental design of the study. (B) Schematic representation showing the injection of a fluorescent tracer (20 kDa FITC‐dextran) into the cisterna magna or brain parenchyma following chronic smoking. (C) Representative brain sections stained for nuclei (DAPI, blue) and FITC‐dextran (green, 20 kDa) showing influx into the brain parenchyma of mice from four groups. Scale bar: 1250 μm. (D) Quantification of FITC‐dextran fluorescence indicating tracer influx distributed in whole brain sections shown in (C). *n* = 6 mice per group. Statistical analysis was performed using one‐way ANOVA. (E) Representative brain sections stained for nuclei (DAPI, blue) and FITC‐dextran (green, 20 kDa) showing clearance from the brain parenchyma of mice from four groups. Scale bar: 1250 μm. (F) Quantification of FITC‐dextran fluorescence indicating tracer residual (efflux) in whole brain sections shown in (E). *n* = 6 mice per group. Statistical analysis was performed using one‐way ANOVA. (G) Schematic showing the evaluation of anterograde CSF flow in PVS of leptomeningeal surface arteries using in vivo two‐photon microscopy following chronic smoking. (H) Perivascular pumping activity assessed in vivo using two‐photon microscopy. The dynamics of CSF were visualized using a 2.5% solution of RITC‐dextran (red, 70 kDa), and the cerebral vasculature was visualized using a 0.025% solution of FITC‐dextran (green, 2000 kDa). Scale bar: 50 μm. (I) Quantification of RITC‐dextran fluorescence distributed in the vision field shown in (H). *n* = 3 mice per group. Statistical analysis was performed using one‐way ANOVA. All data are shown as mean ± SD. *p < 0.05, **p < 0.01. CSF, cerebral spinal fluid; FITC‐dextran, fluorescein isothiocyanate‐dextran; PVS, perivascular space; RITC‐dextran, rhodamine B isothiocyanate‐dextran; SD, standard deviation.

As shown in Figure 1A, mice were randomly distributed into three cohorts, each comprising two groups (a cigarette smoke‐exposed group and a corresponding Sham group). Each cohort underwent either cigarette smoke or air exposure for durations of 4, 8, or 12 weeks, respectively. As previously described, the exposure used a whole‐body smoke exposure system (Yuyan Instruments, Shanghai, China), where mice were exposed daily to the smoke of nine filtered cigarettes from Hongta Tobacco (Yunnan, China) or to filtered room air (Sham) [12]. The exposure chamber used for this experiment measures 40 cm x 40 cm x 40 cm, which was designed to ensure adequate smoke concentration for the animals' exposure. This exposure protocol was maintained for five consecutive days each week throughout the 4 to 12‐week study period [12].

### 2.3 Ex Vivo Evaluation of Glymphatic Influx

Glymphatic influx was evaluated using a 0.5% solution of unfixable fluorescein isothiocyanate‐dextran (FITC‐dextran, 20 kDa, FD20S; Sigma Aldrich) dissolved in artificial CSF. Anesthetized mice were secured in a stereotactic apparatus, and the cisterna magna was surgically exposed. A total volume of 10 μL of CSF tracer was infused into the subarachnoid space at a constant rate of 1 μL/min using a syringe pump (KDS LEGATO 130, RWD Life Science). Thirty minutes after the start of infusion, mice were transcardially perfused with 4% paraformaldehyde (PFA). Brains were harvested, post‐fixed in 4% PFA for 24 h, and sectioned into 100‐μm coronal slices at the hippocampal level. Tracer distribution was visualized using fluorescence microscopy (Olympus BX61) and quantified with ImageJ software (version 1.53, NIH), following previously established protocols [13, 14]. Quantification was performed on six mice per group (*n* = 6).

### 2.4 Ex Vivo Evaluation of Glymphatic Efflux

To investigate the clearance pathway of interstitial metabolites, a 0.5% solution of unfixable fluorescein isothiocyanate‐dextran (FITC‐dextran, 20 kDa, FD20S; Sigma Aldrich) in artificial CSF was stereotactically microinjected into the cerebral cortex. A 33‐gauge stainless steel cannula (Hamilton) was inserted at the following coordinates relative to bregma: posterior −2.00 mm, lateral 1.50 mm, and depth 2.00 mm. Thirty minutes post‐insertion, 2 μL of tracer was infused over 10 min. Mice were transcardially perfused and fixed 1 h post‐infusion. Tracer efflux was quantified using the methodology described above (*n* = 6 mice per group).

### 2.5 In Vivo Evaluation of Perivascular Pumping and Cerebrovascular Pulsatility

Perivascular pumping and cerebrovascular pulsatility were assessed using in vivo two‐photon microscopy as previously described [7, 15, 16]. Briefly, a 3‐mm thinned‐skull cranial window was prepared over the cerebral cortex (1 mm lateral and 0.5 mm posterior to bregma) in anesthetized mice, ensuring dural integrity.

For visualization, 0.5% rhodamine B isothiocyanate‐dextran (R9379, Sigma Aldrich, 70 kDa, RITC‐dextran) was infused intracisternally (1 μL/min for 10 min), and 0.025% fluorescein isothiocyanate‐dextran (FD2000S, Sigma Aldrich, 2000 kDa, FITC‐dextran) was injected intravenously. Imaging commenced 10 min post‐infusion, capturing z‐stacks (512 × 512 pixels, 5 μm steps) from the surface to a depth of 240 μm. Vessels were classified morphologically as previously detailed [7]. Perivascular pumping was quantified by measuring the RITC‐dextran diffusion area around surface arteries and veins (*n* = 3 per group).

Vessel pulsatility was analyzed using 3000 ms X–T line scans orthogonal to the vessel axis at the surface and penetrating levels (100 μm depth). Three vessels per type were randomly selected per mouse. Wall pulsatility (μm × s) was calculated as the absolute area under the diameter–time curve over the 3000 ms epoch using ImageJ and MATLAB (*n* = 3 per group).

### 2.6 Immunohistochemical Staining

Immunohistochemistry (IHC) was performed to detect phosphorylated tau in the hippocampus following established protocols [17, 18, 19]. Sections were incubated overnight at 4°C with rabbit anti‐phospho‐Tau‐S404 (1:250, AP0170; ABclonal) or anti‐phospho‐Tau‐S396 (1:250, AP1028; ABclonal), followed by a biotinylated goat anti‐rabbit secondary antibody (GK500705; Gene Tech) for 1 h at room temperature. Images were acquired using a light microscope (Olympus) and analyzed with ImageJ software (version 1.53; NIH). Phosphorylated tau deposits were quantified within the hippocampal dentate gyrus (DG). Data for each animal represent the mean of three randomly selected coronal sections (*n* = 6 mice per group).

### 2.7 Immunofluorescence

Immunofluorescence staining was performed following established protocols [7, 9]. Tissue sections were incubated with primary antibodies against Doublecortin (1:500; ab18723, Abcam), Synaptophysin (1:500; ab32127, Abcam), PSD95 (1:500; ab18258, Abcam), AQP4 (1:500; 59678, Cell Signaling Technology), GFAP (1:500; 3670, Cell Signaling Technology), and CD31 (1:500; AF3628, R&D Systems), followed by appropriate species‐specific fluorescence‐conjugated secondary antibodies. Images were acquired using a fluorescence microscope (Olympus) and analyzed with ImageJ software (version 1.53; NIH). Fluorescence intensities of SYN, PSD‐95, and DCX were quantified within the hippocampal dentate gyrus (DG). Data represent the mean of three randomly selected coronal sections per animal (*n* = 6 mice per group).

### 2.8 AQP4 Polarization Evaluation

Image and statistical analyses were performed using FIJI, Origin 2021, and GraphPad Prism (version 9.0). Uniform linear pixel intensity adjustments were applied for consistency. AQP4 localization was quantified in FIJI using manually defined ROIs and thresholds in a blinded manner [7, 14]. AQP4 intensity profiles were generated in Origin using 50 μm sampling lines placed orthogonally across large cortical vessels. Perivascular polarization was calculated as previously described [7, 20]. Briefly, the percentage of the area exhibiting immunofluorescence intensity ≥ the median perivascular intensity was determined (AQP4% area). Polarization was defined as:

Polarization=100−AQP4%area.

### 2.9 Western Blot

Western blotting was performed on protein extracts from the hippocampal DG following established protocols. PVDF membranes (Millipore) were blocked with 5% skim milk for 1 h at room temperature and incubated overnight at 4°C with primary antibodies: rabbit anti‐p‐Tau‐S404 (1:250; AP0170, ABclonal), rabbit anti‐p‐Tau‐S396 (1:250; AP1028, ABclonal), and β‐actin (1:1000; TA09, ZSGB‐BIO). Following incubation with corresponding secondary antibodies for 1 h at room temperature, blots were visualized using a ChemiDoc Touch Imaging System and quantified with ImageJ software.

### 2.10 HE and Nissl Staining

Brain tissues fixed in 4% PFA were dehydrated, paraffin‐embedded, and sectioned into 8‐μm coronal slices. Hematoxylin–eosin (HE; G1120, Solarbio) and Nissl (G1432, Solarbio) staining were performed following established protocols [13]. Neurons in the hippocampal DG were identified at 400× magnification based on morphological characteristics: larger soma, round/oval euchromatic nuclei with prominent nucleoli, and abundant cytoplasm (HE); or the presence of cytoplasmic basophilic Nissl bodies (Nissl). Images were acquired using a light microscope (Olympus). Neurons were quantified in the DG region, averaging data from three random coronal sections per animal (*n* = 6 mice per group).

### 2.11 Behavioral Tests

Spatial learning and memory were assessed using the MWM test [21, 22]. The protocol consisted of a 5‐day training phase followed by a spatial probe test on day 6. During training, mice underwent four daily trials (90 s maximum) starting from different quadrants to locate a submerged platform. Mice remaining on the platform for 5 s were removed; those failing to locate it were guided to the platform for 15 s. On day 6, the platform was removed, and mice were released from the opposite quadrant for a 90 s probe trial. Escape latency, path length, swimming velocity, platform crossings, and time in the target quadrant were analyzed using EthoVision XT 13 (Noldus). Quantification was performed on six mice per group (*n* = 6).

Short‐term working memory was evaluated using the NOR test [9, 23]. Mice were habituated to the testing cage for 5 min daily (Days 1–6). On Day 7, mice explored two identical objects for 10 min (familiarization phase). After a 4‐h retention interval, one familiar object was replaced with a novel object (distinct in shape/texture but similar in size), and mice were observed for 5 min. Interaction was defined as the nose pointing toward or touching the object within close proximity. The discrimination ratio was calculated based on the time spent interacting with the novel object relative to the total exploration time. Quantification was performed on six mice per group (*n* = 6).

### 2.12 Echocardiography

Cardiac function was evaluated by transthoracic echocardiography using a Vevo 3100 Imaging System, following established protocols [24]. M‐mode images were acquired from parasternal long‐ and short‐axis views. Left ventricular ejection fraction (EF) and fractional shortening (FS) were measured to assess cardiac performance (*n* = 3 mice per group).

### 2.13 Statistical Analysis

Data analysis and plotting were conducted using GraphPad Prism 9.0. The Shapiro–Wilk test was applied to assess data distribution normality. Statistical differences among groups were analyzed using either one‐way or two‐way ANOVA, followed by Tukey's post hoc test. Statistical significance was set at *p* < 0.05.

## 3 Results

### 3.1 Duration‐Dependent Impairment of Glymphatic Transport Due to Chronic Smoking in Adolescence

Given the critical role of glymphatic CSF‐ISF exchange in neurophysiology [10]. We first investigated whether chronic smoking impairs glymphatic influx. Analysis revealed that tracer penetration was significantly reduced in smoking groups compared to Sham controls. Importantly, this impairment exacerbated in a duration‐dependent manner, with severity escalating alongside prolonged smoke exposure (Figure 1B–D and Figure S1A–C).

Next, we evaluated metabolic waste clearance within the brain parenchyma via intracortical tracer injection. Consistent with influx findings, the capacity for waste clearance was significantly reduced in smoking groups relative to Sham controls. This impairment of glymphatic efflux also intensified with prolonged smoke exposure (Figure 1E,F and Figure S1D,E).

Finally, we assessed perivascular pumping—the mechanism driving CSF flow along low‐resistance paravascular channels [25]—using in vivo two‐photon microscopy. Dual‐tracer imaging (RITC‐dextran for CSF; FITC‐dextran for vasculature) allowed for the simultaneous visualization of leptomeningeal surface vessels and CSF dynamics. Analysis indicated that perivascular pumping activity was significantly diminished in smoking groups compared to Sham controls. Notably, the severity of this dysfunction escalated with increasing duration of smoke exposure (Figure 1G–I, Figure S1F, and Videos [Link], [Link], [Link], [Link], [Link], [Link]).

### 3.2 Chronic Smoking is Associated With Increased Phosphorylated Tau in the Hippocampal DG

**FIGURE 2:**
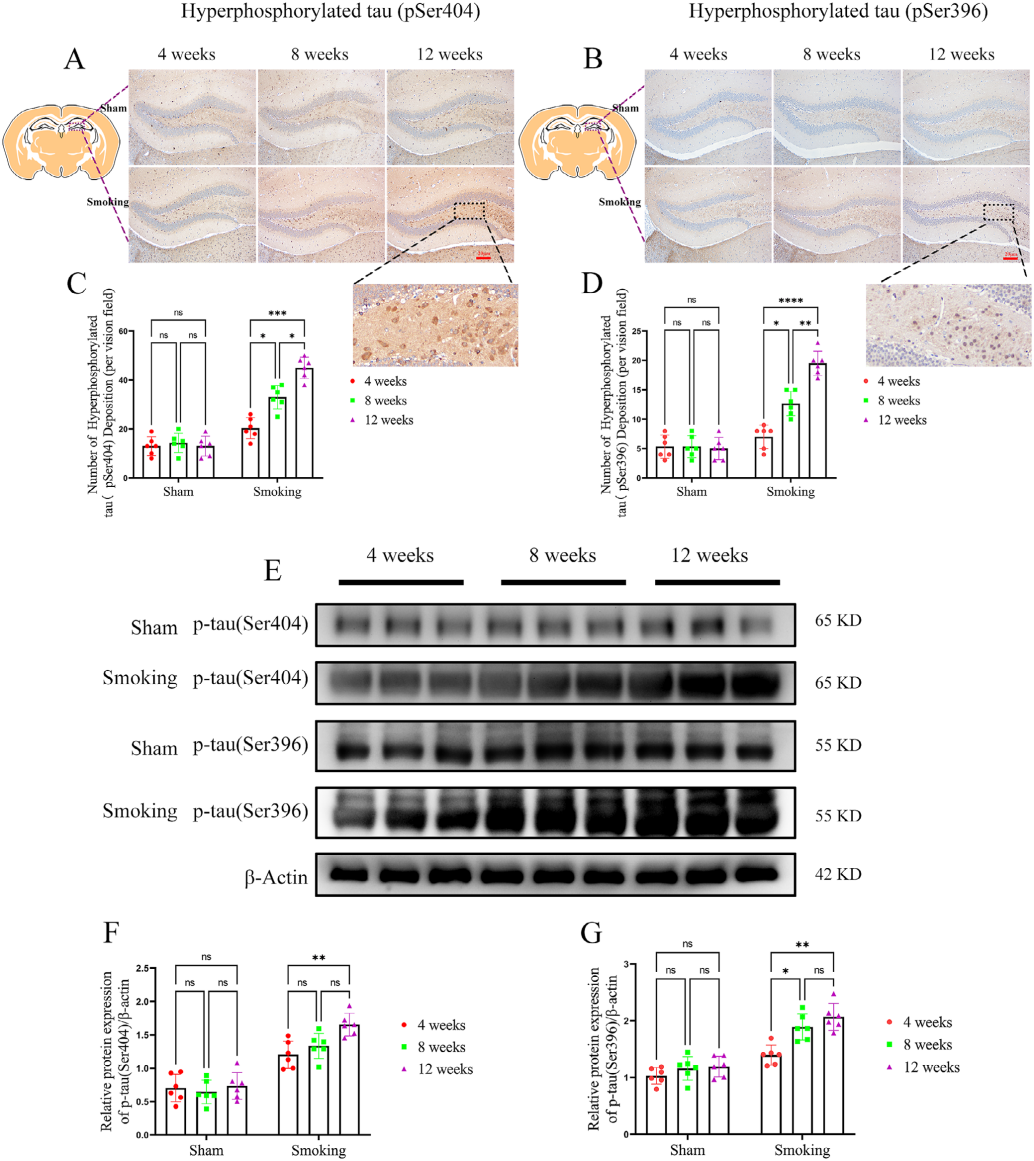
Impairment of glymphatic pathway function promotes the development of tauopathy following chronic smoking. (A) Representative immunohistochemical images of phosphorylated tau (pSer404) in the hippocampal DG region of mice following chronic smoking exposure. Scale bar, 20 μm. (B) Representative immunohistochemical images of phosphorylated tau (pSer396) in the hippocampal DG region of mice following chronic smoking exposure. Scale bar, 20 μm. (C) Quantitative analysis of phosphorylated tau (pSer404) accumulation. *n* = 6 mice per group. Statistical analysis was performed using one‐way ANOVA. (D) Quantitative analysis of phosphorylated tau (pSer396) accumulation. *n* = 6 mice per group. Statistical analysis was performed using one‐way ANOVA. (E) Representative Western blots of phosphorylated tau (pSer404) and phosphorylated tau (pSer396). (F and G) Quantification of relative protein expression normalized to the optical density of β‐Actin. *n* = 6 mice per group. Statistical analysis was performed using one‐way ANOVA. All data are shown as mean ± SD. *p < 0.05, **p < 0.01, ***p < 0.001, ****p < 0.0001. DG, dentate gyrus; SD, standard deviation.

Neurofibrillary tangles composed of hyperphosphorylated tau protein are implicated in cognitive decline associated with central nervous system disorders [8, 9, 20]. To determine whether chronic smoking was associated with tau phosphorylation in the hippocampal DG, immunohistochemical staining with anti‐pSer404 tau and anti‐pSer396 tau antibodies was performed. As shown in the representative images and quantitative analysis (Figure 2A–D), chronic smoking exposure increased phosphorylated tau signals, including pSer404 and pSer396, in the DG region compared with the corresponding Sham groups. Western blot analysis further supported these findings, showing increased protein levels of pSer404 and pSer396 in the smoking groups compared with controls (Figure 2E–G). Importantly, this aberrant accumulation likely reflects not only the impaired glymphatic clearance of extracellular tau but also the enhanced intracellular production and promotion of tau phosphorylation driven by smoking‐induced neurotoxicity.

### 3.3 Chronic Smoking Impairs Synaptogenesis and Neurogenesis in the Hippocampal DG Region

**FIGURE 3:**
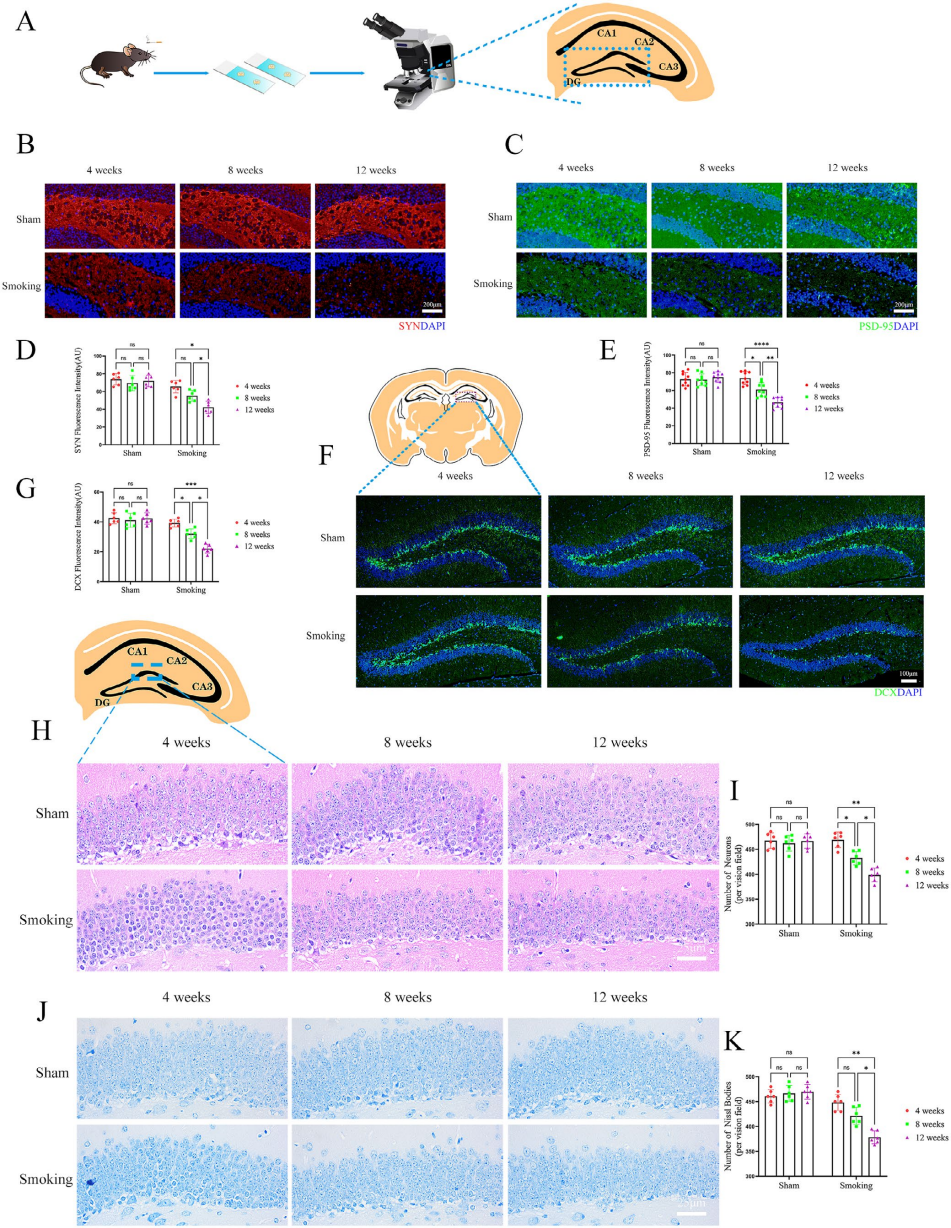
Chronic smoking impairs synaptogenesis and neurogenesis in the hippocampal DG region. (A) Schematic representation of the immunofluorescence staining method and procedure. (B) Representative immunofluorescence images of SYN (red) in the DG region of mice following chronic smoking exposure. Scale bar: 200 μm. (C) Representative immunofluorescence images of PSD‐95 (green) in the DG region of mice following chronic smoking exposure. Scale bar: 200 μm. (D) Quantitative analysis of SYN fluorescence intensity. *n* = 6 mice per group. Statistical analysis was performed using one‐way ANOVA. (E) Quantitative analysis of PSD‐95 fluorescence intensity. *n* = 6 mice per group. Statistical analysis was performed using one‐way ANOVA. (F) Representative immunofluorescence images of DCX (green) in the DG region of mice following chronic smoking exposure. Scale bar: 100 μm. (G) Quantitative analysis of DCX fluorescence intensity. *n* = 6 mice per group. Statistical analysis was performed using one‐way ANOVA. (H) Representative HE images of neurons in the DG region of mice following chronic smoking exposure. Scale bar: 25 μm. (I) Quantitative analysis of neurons shown in (H). *n* = 6 mice per group. Statistical analysis was performed using one‐way ANOVA. (J) Representative Nissl staining images in the DG region of mice following chronic smoking exposure. Scale bar: 25 μm. (K) Quantitative analysis of Nissl bodies shown in (J). *n* = 6 mice per group. Statistical analysis was performed using one‐way ANOVA. All data are shown as mean ± SD. *p < 0.05, **p < 0.01, ***p < 0.001, ****p < 0.0001. DCX, doublecortin; DG, dentate gyrus; HE, hematoxylin and eosin; PSD‐95, postsynaptic density protein‐95; SD, standard deviation; SYN, synaptophysin.

Hippocampal synaptic dysfunction is a key contributor to cognitive deficits. Synaptophysin (SYN) and PSD‐95 are particularly susceptible to p‐tau toxicity, and their loss correlates with cognitive impairment [26, 27, 28]. Immunofluorescence analysis revealed that chronic smoking significantly reduced SYN and PSD‐95 expression in the hippocampal dentate gyrus (DG) compared to Sham controls, indicating impaired synaptogenesis (Figure 3A–E). Hippocampal neurogenesis is essential for learning and memory [29, 30]. Using doublecortin (DCX) as a marker for immature neurons [31], we observed that chronic smoking reduced DCX expression in the DG relative to Sham groups (Figure 3F,G). Histopathological changes were further evaluated using HE and Nissl staining. Nissl bodies, which reflect active protein synthesis, are sensitive markers of neuronal injury [32]. As shown in Figure 3H–K, chronic smoking led to a significant reduction in neuronal density and Nissl body count in the DG compared to Sham controls.

### 3.4 Chronic Smoking Results in Cognitive Dysfunction, Affecting Spatial Learning, Memory, and Working Memory

**FIGURE 4:**
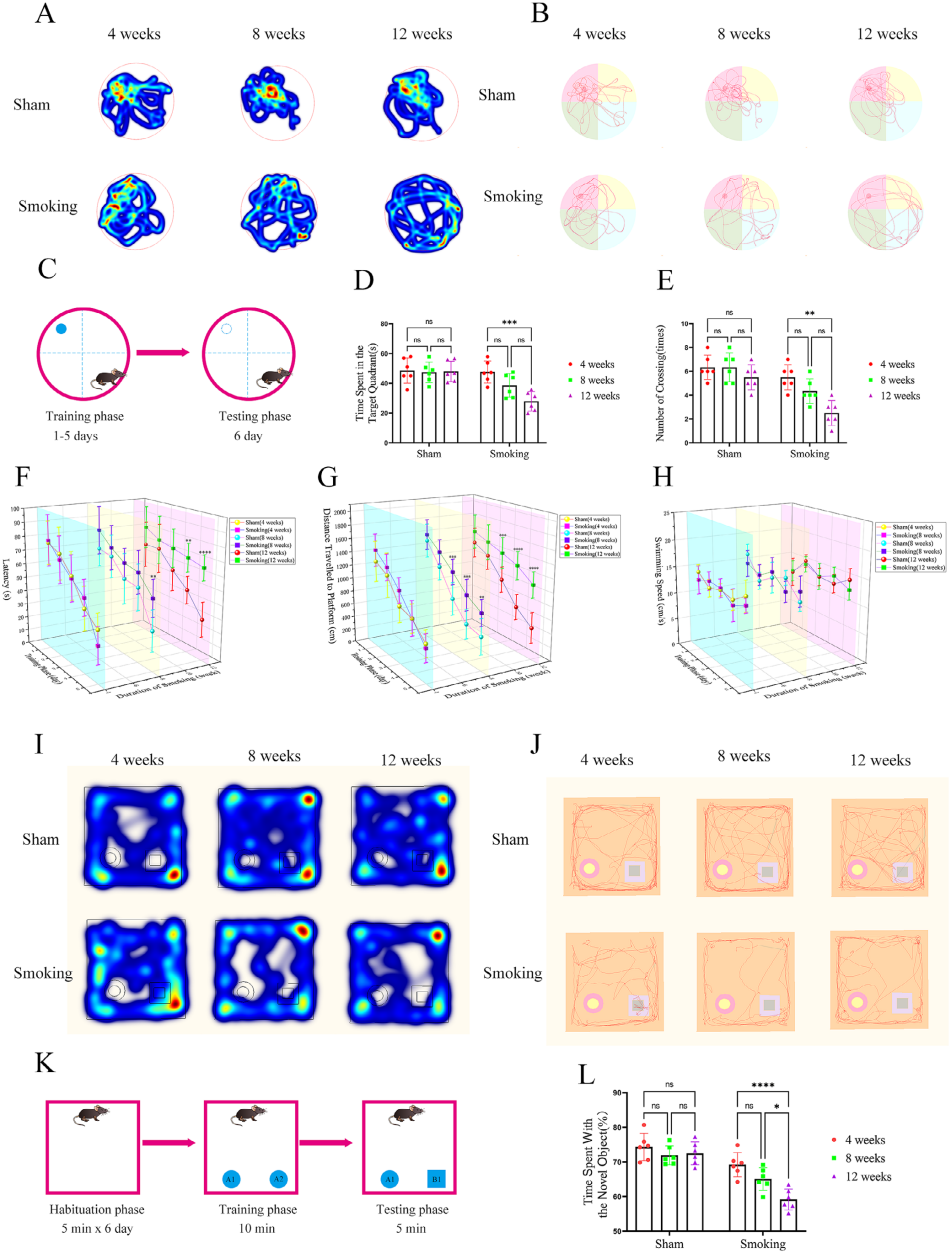
Chronic smoking induces cognitive dysfunction, impairing spatial learning, memory, and working memory. (A) Representative thermal imaging during the probe trial of the MWM test. (B) Representative imaging of swimming trajectories in the MWM test. (C) Schematic representation of the methodology and process for the Morris Water Maze (MWM) test. (D–H) Analysis of MWM test data assessing spatial learning and memory abilities in mice subjected to chronic smoking exposure. *n* = 6 mice per group. Statistical analysis for (D–F) was performed using two‐way ANOVA. Statistical analysis for (G and H) was performed using one‐way ANOVA. (I) Representative thermal imaging during the probe trial of the NOR test. (J) Representative imaging of exploratory behavior in the NOR test. (K) Schematic representation of the methodology and process for the Novel Object Recognition (NOR) test. (L) Analysis of time spent with the novel object assessing short‐term working memory abilities in mice subjected to chronic smoking exposure. *n* = 6 mice per group. Statistical analysis was performed using one‐way ANOVA. All data are shown as mean ± SD. *p < 0.05, **p < 0.01, ***p < 0.001, ****p < 0.0001. MWM test, morris water maze test; NOR test, novel object recognition test; SD, standard deviation.

To evaluate cognitive competence, neurobehavioral assessments were conducted using the Morris Water Maze (MWM) and Novel Object Recognition (NOR) tests. During the MWM training phase (Figure 4C), mice exposed to chronic smoking exhibited significantly increased escape latency and path length compared to Sham controls. Swimming velocities were comparable across groups, ruling out motor deficits as a confounding factor (Figure 4A,B,F–H). In the probe trial, smoking mice showed reduced time in the target quadrant and fewer platform crossings relative to controls (Figure 4D,E). Furthermore, the NOR test (Figure 4K) revealed working memory impairments, with smoking mice spending significantly less time exploring the novel object (Figure 4I,J,L). Collectively, these findings indicate that chronic smoking induces a duration‐dependent deterioration in spatial learning, memory, and working memory.

### 3.5 Mechanisms of Glymphatic System Impairment by Chronic Smoking

**FIGURE 5:**
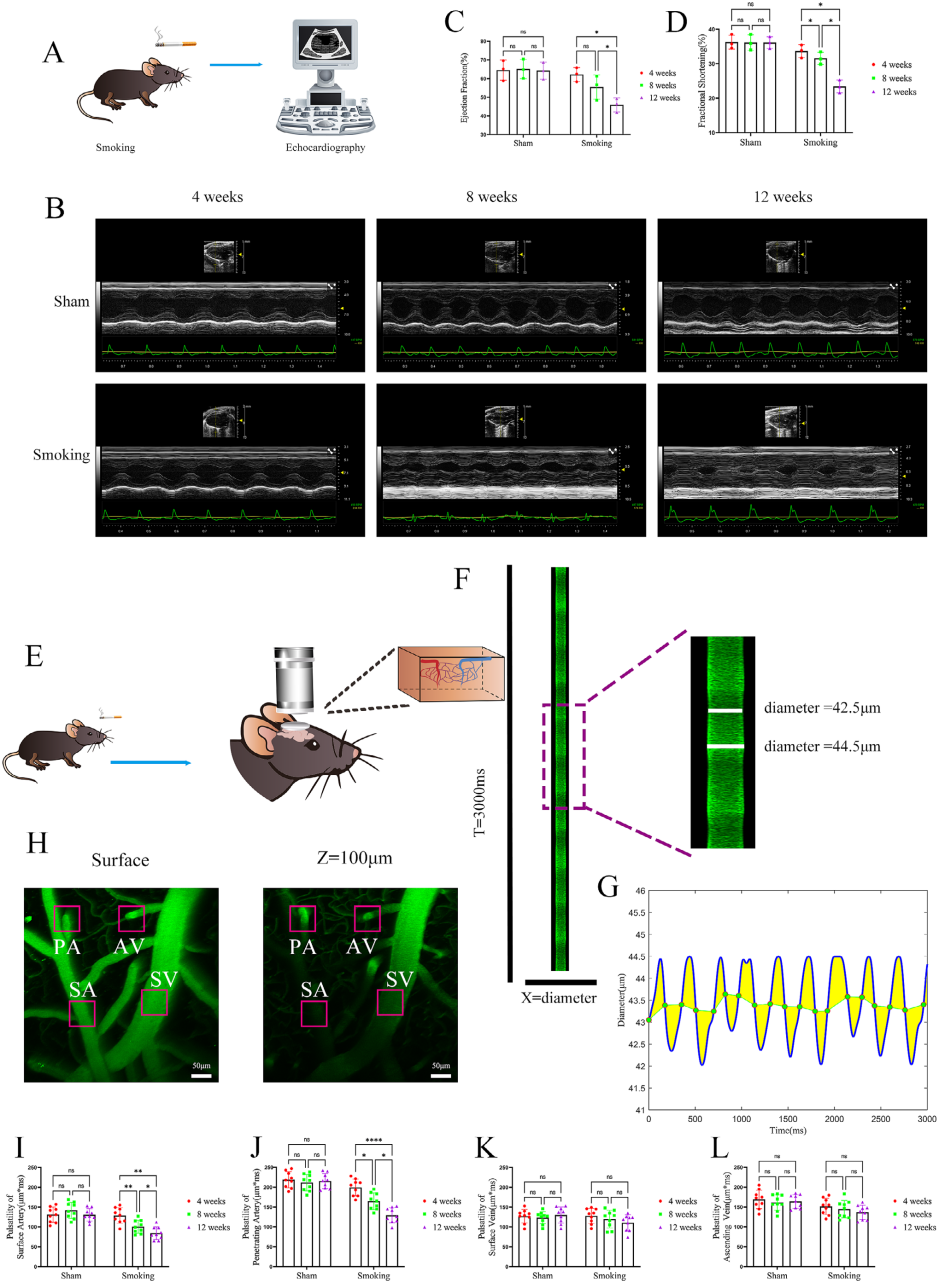
Chronic smoking impairs cardiac contractile function and cerebral vascular pulsatility in mice. (A) Schematic representation of the evaluation of cardiac contractile function using echocardiography. (B) Representative echocardiographic images of mice following chronic smoking exposure. (C) Quantification of left ventricular EF in mice following chronic smoking exposure. *n* = 3 mice per group. Statistical analysis was performed using one‐way ANOVA. (D) Quantification of left ventricular FS in mice following chronic smoking exposure. *n* = 3 mice per group. Statistical analysis was performed using one‐way ANOVA. (E) Schematic representation of the evaluation of cerebral vascular pulsatility using in vivo two‐photon microscopy following chronic smoking exposure. (F) X–T line scans were generated orthogonal to the vessel axis. (G) Vessel diameter was measured and plotted as a function of time. (H) In vivo two‐photon microscopy imaging at different depths (surface and 100 μm below the surface) to distinguish various vessel types. Scale bar = 50 μm. (I) Statistical analysis of SA pulsatility in different groups of mice. *n* = 3 mice per group. Statistical analysis was performed using one‐way ANOVA. (J) Statistical analysis of PA pulsatility in different groups of mice. *n* = 3 mice per group. Statistical analysis was performed using one‐way ANOVA. (K) Statistical analysis of SV pulsatility in different groups of mice. *n* = 3 mice per group. Statistical analysis was performed using one‐way ANOVA. (L) Statistical analysis of AV pulsatility in different groups of mice. *n* = 3 mice per group. Statistical analysis was performed using one‐way ANOVA. The data in (G) are from individual representative samples; The data in (C and D) and (I–L) are presented as mean ± SD. *p < 0.05, **p < 0.01, ****p < 0.0001. AV, ascending veins; EF, ejection fraction; FS, fractional shortening; PA, penetrating arteries; SA, surface arteries; SD, standard deviation; SV, surface veins.

Bulk flow of CSF in the perivascular space (PVS) in the murine brain is partly driven by arterial pulsatility arising from the cardiac systolic pressure wave [8, 33, 34]. To explore the effects of chronic smoking on cardiac contractile function, echocardiography was utilized to evaluate the left ventricular EF and FS. As illustrated in the representative images (Figure 5A–D and Figure S2A–C), chronic smoking exposure results in a marked decrease in EF and FS across varying degrees compared to the corresponding Sham groups, indicating compromised cardiac contractile function.

Next, we investigated the pulsatility of the cerebrovascular wall using in vivo 2‐photon microscopy. Visualization of the cerebral vasculature was accomplished via intra‐arterial administration of 2000 kDa FITC‐Dextran, observed through a thin‐skull cranial window setup (Figure 5E–H). We recorded small alterations in the diameter of penetrating arteries and ascending veins up to 150 mm beneath the cortical surface through high‐frequency line scanning. Consistent with methods outlined previously, we calculated vascular pulsatility values from diameter‐time (X‐T) plots (Figure 5F,G), which were based on the cyclic expansion of vessel diameter during each cardiac cycle, analyzed over a 3000‐millisecond period [7]. Our observations indicate that chronic smoking exposure results in time‐related varying degrees of reduction in the pulsatility of both surface and penetrating arteries in mice, in comparison with their corresponding Sham groups. Meanwhile, no smoking‐related alterations were detected in the pulsatility of surface veins and ascending veins (Figure 5I–L and Figure S2D).

**FIGURE 6:**
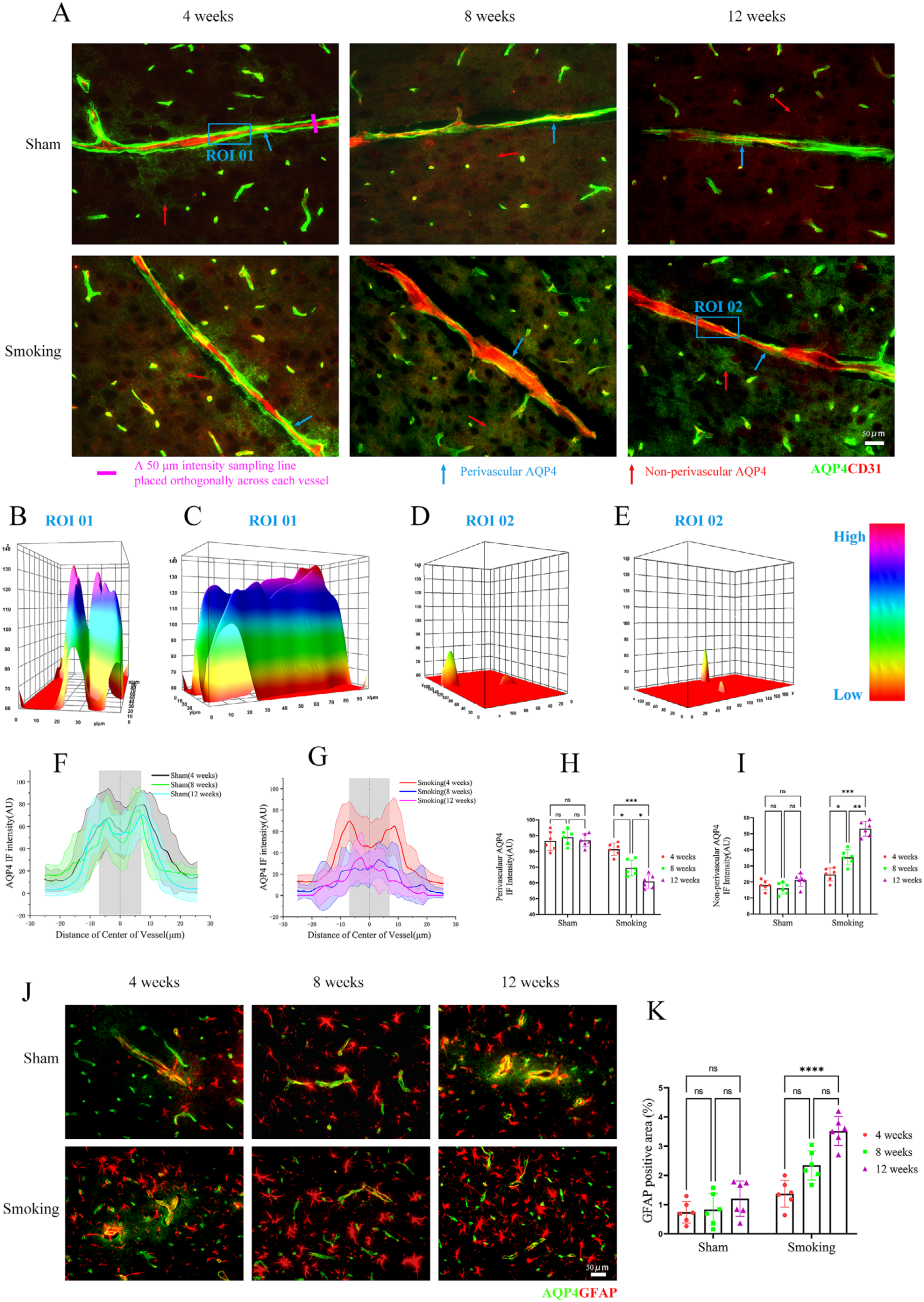
Chronic smoking induces loss of perivascular AQP4 in the mouse brain. (A) Coimmunofluorescence staining for AQP4 (green) and CD31 (red) in the mouse brain following chronic smoking exposure. Scale bar: 50 μm. (B–E) 3D surface plot reconstruction of the ROI showing the distribution of perivascular AQP4. (F and G) Quantification of AQP4 immunofluorescence projections across large cortical vessels in the mouse brain. Graph shows solid lines for mean values with SE represented as shading. *n* = 6 mice per group. (H) Quantification of AQP4 expression surrounding large cortical vessels in the mouse brain. *n* = 6 mice per group. Statistical analysis was performed using one‐way ANOVA. (I) Quantification of non‐perivascular AQP4 expression in the mouse brain. *n* = 6 mice per group. Statistical analysis was performed using one‐way ANOVA. (J) Immunofluorescence staining for AQP4 (green) and GFAP (red) in the mouse brain following chronic smoking exposure. Scale bar: 50 μm. (K) Quantification of GFAP positive area in the field. *n* = 6 mice per group. Statistical analysis was performed using one‐way ANOVA. The data in (B–E) are from individual representative samples; The data in (F and G) are presented as mean ± SE; The data in (H) and (J) are presented as mean ± SD. *p < 0.05, **p < 0.01, ***p < 0.001, ****p < 0.0001. AQP4, aquaporin‐4; GFAP, glial fibrillary acidic protein; ROI, region of interest; SD, standard deviation; SE, standard error.

AQP4 is strategically localized to perivascular astrocytic endfeet to reduce resistance and enhance CSF‐ISF exchange [8, 9, 10]. Loss of this polarization is a hallmark of glymphatic dysfunction [7, 8, 9]. Using AQP4 and CD31 co‐staining (Figure 6A), we found that chronic smoking altered AQP4 spatial distribution, causing a duration‐dependent reduction in perivascular AQP4 and a concurrent increase in non‐perivascular AQP4 (Figure 6B–I and Figure S3A–D). Since reactive astrogliosis is closely associated with AQP4 depolarization [7, 8, 9], we further observed that chronic smoking elicited a duration‐dependent increase in reactive astrogliosis relative to Sham groups (Figure 6J,K).

Collectively, the mechanisms by which chronic smoking exposure leads to impaired glymphatic transport partly involve compromised cardiac ejection function, diminished arterial pulsatility in the brain, and the loss of perivascular AQP4.

## 4 Discussion

While the pulmonary and cardiovascular risks of smoking are established, growing evidence links tobacco use to CNS impairments, including accelerated cognitive decline and neurodegenerative disorders [32]. Rodent models demonstrate that smoke exposure exacerbates AD‐related pathologies, such as Aβ accumulation, neuroinflammation, and tau phosphorylation, and is implicated in other protein misfolding disorders [1, 35].

Pulsatile flow at the brain surface is thought to play a crucial role in clearing waste from the brain parenchyma [25, 36]. Anterograde flow of CSF in the PVS of leptomeningeal surface arteries, driven by perivascular pumping mechanisms, facilitates the diffusion of solutes throughout the brain [34]. This flow subsequently enters the brain parenchyma via the PVS of penetrating arteries, representing a microcosmic model of the glymphatic system's fluid transport function. Following this, CSF‐ISF exchange is hypothesized to operate within the brain parenchyma [8]. Direct physical connections between the PVS of ascending veins and leptomeningeal vessels further support the clearance of solutes and metabolic waste via bulk CSF flow along the brain surface [25]. In this study, 4–12 weeks of cigarette smoke exposure resulted in a duration‐dependent reduction in CSF bulk flow and tracer distribution along surface paravascular spaces, reflecting impaired perivascular dynamics. While PVS size variations are a potential confounder, these structural changes likely alter tracer diffusion and contribute to the observed signal reduction.

Efficient paravascular CSF‐ISF exchange relies critically on the polarized expression of AQP4 on astrocytic endfeet. AQP4 depolarization reduces CSF influx and clearance [7, 37], while its complete absence significantly diminishes glymphatic function [4, 9, 20, 34, 38]. Reactive astrogliosis and the loss of perivascular AQP4 polarization are common features of brain injury [9], promoting the aggregation of neurotoxic proteins like Aβ and tau. CSF bulk flow is proposed to be driven by arterial pulsatility derived from the cardiac cycle [8, 33, 34, 36]. Pressure waves transmitted along major and penetrating arteries propel CSF into the neuropil via periarterial spaces [39]; consequently, diminished pulsatility associated with vascular stiffening (e.g., aging, hypertension) compromises clearance [36]. In this study, 4–12 weeks of cigarette smoke exposure significantly reduced cardiac output (decreased EF and FS), leading to diminished pulsatility in cerebral surface and penetrating arteries. This impairment may be further exacerbated by smoke‐induced atherosclerosis and altered vascular compliance [40].

AQP4 is predominantly localized to astrocytic endfeet, which is believed to facilitate CSF‐ISF exchange and efficient glymphatic transport. However, chronic smoking induces a duration‐dependent shift in AQP4 distribution from perivascular to non‐perivascular regions. Several mechanisms likely contribute to this loss of polarization. First, smoking‐induced reactive astrogliosis involves morphological changes that may disrupt normal perivascular AQP4 anchoring. Second, smoking may alter the ratio of AQP4 isoforms; specifically, the upregulation of the non‐polarized AQP4‐M1 isoform, relative to the highly polarized AQP4‐M23, could drive AQP4 mislocalization. Furthermore, long‐term smoking impairs blood–brain barrier integrity and reduces arterial pulsatility, both of which are essential for maintaining proper AQP4 distribution [41, 42]. Collectively, AQP4 depolarization, reactive astrogliosis, and reduced arterial pulsatility may act together to impair glymphatic transport [7, 9]. The accumulation of hyperphosphorylated tau observed in our study is likely a synergistic outcome: smoking toxicity directly promotes intracellular tau phosphorylation, while concurrent glymphatic failure prevents the efficient clearance of extracellular tau, creating a toxic microenvironment that further exacerbates intracellular tangle formation.

It is critical to acknowledge that glymphatic transport is a highly complex and multifactorial process driven not only by arterial pulsatility but also by respiratory cycles, vasomotor waves, and sleep–wake states [8, 34]. In the context of chronic smoking, we conceptualize a synergistic “multiple‐hit” model rather than a singular causal pathway. While toxic smoke constituents independently induce local parenchymal injury such as AQP4 depolarization, the concurrent systemic impairment of cardiac ejection acts as a fundamental mechanical bottleneck [33, 43]. This cardiovascular deterioration severely restricts the kinetic force required for initial periarterial CSF influx, working in tandem with local damage to precipitate glymphatic failure.

This study has several limitations. First, our whole‐body exposure model utilized a complex mixture of combustible cigarette smoke to maximize clinical relevance. Consequently, we assessed the overall effect of smoke exposure and could not isolate the direct neurotoxic effects of specific components, such as nicotine, from the secondary systemic effects driven by other combustible constituents, including systemic inflammation, hypoxia, and endothelial injury. Rather than acting merely as confounding factors, these systemic and cardiovascular consequences may represent important upstream contributors to the impaired cardiac ejection and diminished arterial pulsatility observed in our study [44]. Future studies using isolated nicotine or component‐specific exposure models will be essential to dissect these mechanisms. Second, our histological analyses focused mainly on the hippocampal DG because the DG contains the subgranular zone and supports adult hippocampal neurogenesis. Therefore, the observed 10%–20% reduction in DG neuronal density should be interpreted as a cumulative effect involving suppressed neurogenesis, impaired maturation/integration of newborn granule cells, and smoking/glymphatic dysfunction‐related neuronal injury, rather than acute mature neuronal loss alone. We also acknowledge that the absence of CA1/CA3 quantification limits broader anatomical interpretation.

## 5 Conclusions

This study demonstrates that chronic cigarette smoke exposure in adolescent mice induces duration‐dependent glymphatic dysfunction. This impairment leads to the accumulation of phosphorylated tau in the hippocampus, which suppresses neurogenesis and synaptogenesis in the dentate gyrus, ultimately resulting in cognitive deficits.

## Author Contributions

Chuanxiang Lv and Li Bie conceived and designed the study. Chengyu Xia, Zean Li, and Tao Liu wrote the manuscript, which was revised by Xinran Zhang and Rongcai Jiang, approved by all the authors. Tao Liu, Shiying Dong, Zhuang Sha, and Yu Qian performed model preparation. Chuanxiang Lv, Chengyu Xia, and Yu Qian performed Two‐photon microscopy experiment. Mingqi Liu and Zean Li performed CSF tracer infusions. Kang Zhang performed immunostaining. Kang Zhang and Xinran Zhang performed behavioral tests. Rongcai Jiang and Li Bie performed statistical analyses of the data.

## Funding

This work was supported by Science and Technology Development Plan Project of Jilin Province, China (Nos. 20230204094YY, 2022LC093, YDZJ202201ZYTS001) and National Natural Science Foundation of China (Grant no. 82201518 to Yu Qian).

## Ethics Statement

All animal experiments were conducted in accordance with the National Institutes of Health Guide for the Care and Use of Laboratory Animals and were approved by the Animal Ethics Committee of The First Hospital of Jilin University (Approval No. 20250222–21).

## Conflicts of Interest

The authors declare no conflicts of interest.

## Supporting information


**Figure S1:** Regional evaluation of glymphatic transport and 3D volumetric rendering of perivascular space dynamics. (A) Representative anatomical atlas‐mapped images illustrating glymphatic influx (left) and glymphatic efflux (right) in the Sham and Smoking groups at the 12‐week time point. (B) Quantification of FITC‐dextran fluorescence indicating tracer influx distributed in the cortical region. *n* = 6 mice per group. Statistical analysis was performed using one‐way ANOVA. (C) Quantification of FITC‐dextran fluorescence indicating tracer influx distributed in the basal region. *n* = 6 mice per group. Statistical analysis was performed using one‐way ANOVA. (D) Quantification of FITC‐dextran fluorescence residual in the cortical region after intraparenchymal injection (efflux). *n* = 6 mice per group. Statistical analysis was performed using one‐way ANOVA. (E) Quantification of FITC‐dextran fluorescence residual in the hippocampus after intraparenchymal injection (efflux). *n* = 6 mice per group. Statistical analysis was performed using one‐way ANOVA. (F) Advanced 3D volumetric rendering (left and middle) and orthogonal views (right) of perivascular space dynamics assessed in vivo using two‐photon microscopy in Sham and Smoking mice at 12 weeks. The cerebral vasculature was visualized using a 0.025% solution of FITC‐dextran (green, 2000 kDa), and CSF dynamics were visualized using a 2.5% solution of RITC‐dextran (red, 70 kDa). Scale bar: 100 μm. All data are shown as mean ± SD. *p < 0.05, *p* < 0.01, ***p < 0.001. FITC‐dextran, fluorescein isothiocyanate‐dextran; CSF, cerebral spinal fluid; RITC‐dextran, rhodamine B isothiocyanate‐dextran; SD, standard deviation.

**Figure S2:** Evaluation of cardiac function and 3D reconstruction of cerebrovascular morphology. (A) Quantification of beats per minute (BPM) in mice following chronic smoking exposure (*n* = 3 mice per group). Statistical analysis was performed using one‐way ANOVA. (B) Quantification of left ventricular ejection fraction (EF) in mice following chronic smoking exposure (*n* = 3 mice per group). Statistical analysis was performed using one‐way ANOVA. (C) Quantification of left ventricular fractional shortening (FS) in mice following chronic smoking exposure (*n* = 3 mice per group). Statistical analysis was performed using one‐way ANOVA. (D) Different angles of 3D reconstruction of vessel morphology showing the spatial relationship of surface arteries (SA), surface veins (SV), penetrating arteries (PA), and ascending veins (AV). Green indicates vessel morphology; blue lines indicate 3D‐reconstructed region boundaries. All data are shown as mean ± SD. *p < 0.05, **p < 0.01. AV, ascending veins; EF, ejection fraction; FS, fractional shortening; PA, penetrating arteries; SA, surface arteries; SD, standard deviation; SV, surface veins.

**Figure S3:** Alterations in perivascular AQP4 distribution following chronic smoking. (A–C) Quantification of AQP4 immunofluorescence intensity profiles across large cortical vessels in the mouse brain following 4 weeks (A), 8 weeks (B), and 12 weeks (C) of exposure. The graph shows solid lines for mean values with standard error (SE) represented as shading. (D) Quantification of AQP4 polarization in the mouse brain following chronic smoking exposure. *n* = 6 mice per group. Statistical analysis was performed using one‐way ANOVA. Data in (D) are presented as mean ± SD. *p < 0.05, ****p < 0.0001.

**Video S1:** In vivo perivascular pumping in the Sham group at 4 weeks. Representative two‐photon microscopy time‐lapse imaging illustrates perivascular pumping activity in a control mouse following 4 weeks of air exposure. CSF dynamics are visualized using RITC‐dextran (red, 70 kDa), and cerebral vasculature is visualized using FITC‐dextran (green, 2000 kDa).

**Video S2:** In vivo perivascular pumping in the Sham group at 8 weeks. Representative two‐photon microscopy time‐lapse imaging illustrates perivascular pumping activity in a control mouse following 8 weeks of air exposure. CSF dynamics are visualized using RITC‐dextran (red, 70 kDa), and cerebral vasculature is visualized using FITC‐dextran (green, 2000 kDa).

**Video S3:** In vivo perivascular pumping in the Sham group at 12 weeks. Representative two‐photon microscopy time‐lapse imaging illustrates perivascular pumping activity in a control mouse following 12 weeks of air exposure. CSF dynamics are visualized using RITC‐dextran (red, 70 kDa), and cerebral vasculature is visualized using FITC‐dextran (green, 2000 kDa).

**Video S4:** In vivo perivascular pumping in the Smoking group at 4 weeks. Representative two‐photon microscopy time‐lapse imaging illustrates perivascular pumping activity in a mouse following 4 weeks of chronic cigarette smoke exposure. CSF dynamics are visualized using RITC‐dextran (red, 70 kDa), and cerebral vasculature is visualized using FITC‐dextran (green, 2000 kDa).

**Video S5:** In vivo perivascular pumping in the Smoking group at 8 weeks. Representative two‐photon microscopy time‐lapse imaging illustrates perivascular pumping activity in a mouse following 8 weeks of chronic cigarette smoke exposure. CSF dynamics are visualized using RITC‐dextran (red, 70 kDa), and cerebral vasculature is visualized using FITC‐dextran (green, 2000 kDa).

**Video S6:** In vivo perivascular pumping in the Smoking group at 12 weeks. Representative two‐photon microscopy time‐lapse imaging illustrates perivascular pumping activity in a mouse following 12 weeks of chronic cigarette smoke exposure. CSF dynamics are visualized using RITC‐dextran (red, 70 kDa), and cerebral vasculature is visualized using FITC‐dextran (green, 2000 kDa). FITC‐dextran, fluorescein isothiocyanate‐dextran; RITC‐dextran, rhodamine B isothiocyanate‐dextran.

## Data Availability

The data that support the findings of this study are available from the corresponding author upon reasonable request.
