# Supplementary figures and images for: Chronic Smoking Impairs Glymphatic Transport and Cognitive Function in Adolescent Mice Through Cardiac, Vascular, and Perivascular Aquaporin‐4 Mechanisms
Source: CNS Neurosci Ther. 2026 Aug 3;32(8):e71040. doi: 10.1002/cns.71040 (PMC13430975; doi:10.1002/cns.71040)

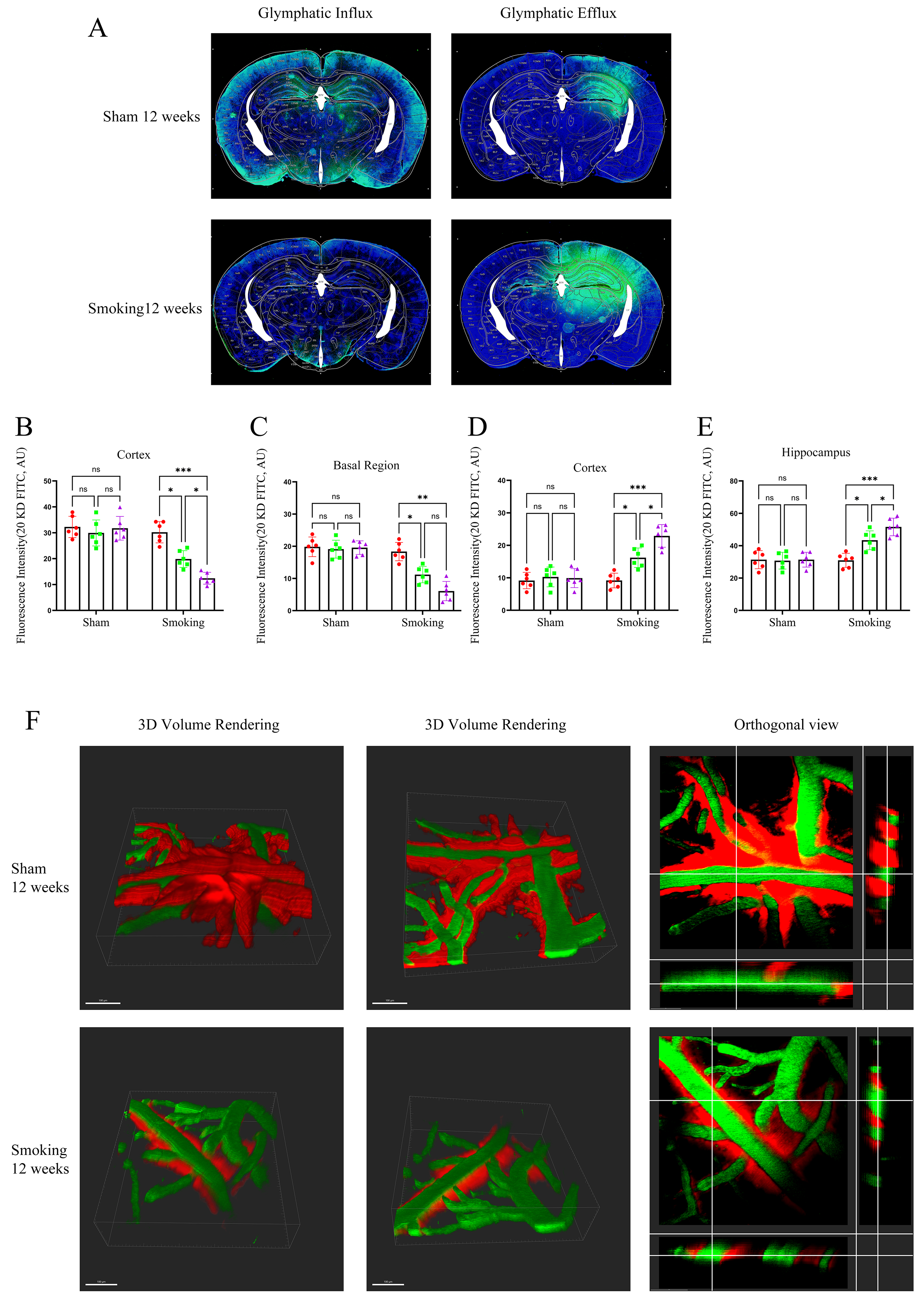

Supplement: Supplementary file 1 — Figure S1: Regional evaluation of glymphatic transport and 3D volumetric rendering of perivascular space dynamics. (A) Representative anatomical atlas‐mapped images illustrating glymphatic influx (left) and glymphatic efflux (right) in the Sham and Smoking groups at the 12‐week time point. (B) Quantification of FITC‐dextran fluorescence indicating tracer influx distributed in the cortical region. n = 6 mice per group. Statistical analysis was performed using one‐way ANOVA. (C) Quantification of FITC‐dextran fluorescence indicating tracer influx distributed in the basal region. n = 6 mice per group. Statistical analysis was performed using one‐way ANOVA. (D) Quantification of FITC‐dextran fluorescence residual in the cortical region after intraparenchymal injection (efflux). n = 6 mice per group. Statistical analysis was performed using one‐way ANOVA. (E) Quantification of FITC‐dextran fluorescence residual in the hippocampus after intraparenchymal injection (efflux). n = 6 mice per group. Statistical analysis was performed using one‐way ANOVA. (F) Advanced 3D volumetric rendering (left and middle) and orthogonal views (right) of perivascular space dynamics assessed in vivo using two‐photon microscopy in Sham and Smoking mice at 12 weeks. The cerebral vasculature was visualized using a 0.025% solution of FITC‐dextran (green, 2000 kDa), and CSF dynamics were visualized using a 2.5% solution of RITC‐dextran (red, 70 kDa). Scale bar: 100 μm. All data are shown as mean ± SD. *p < 0.05, p < 0.01, ***p < 0.001. FITC‐dextran, fluorescein isothiocyanate‐dextran; CSF, cerebral spinal fluid; RITC‐dextran, rhodamine B isothiocyanate‐dextran; SD, standard deviation. [file CNS-32-e71040-s003.tif]

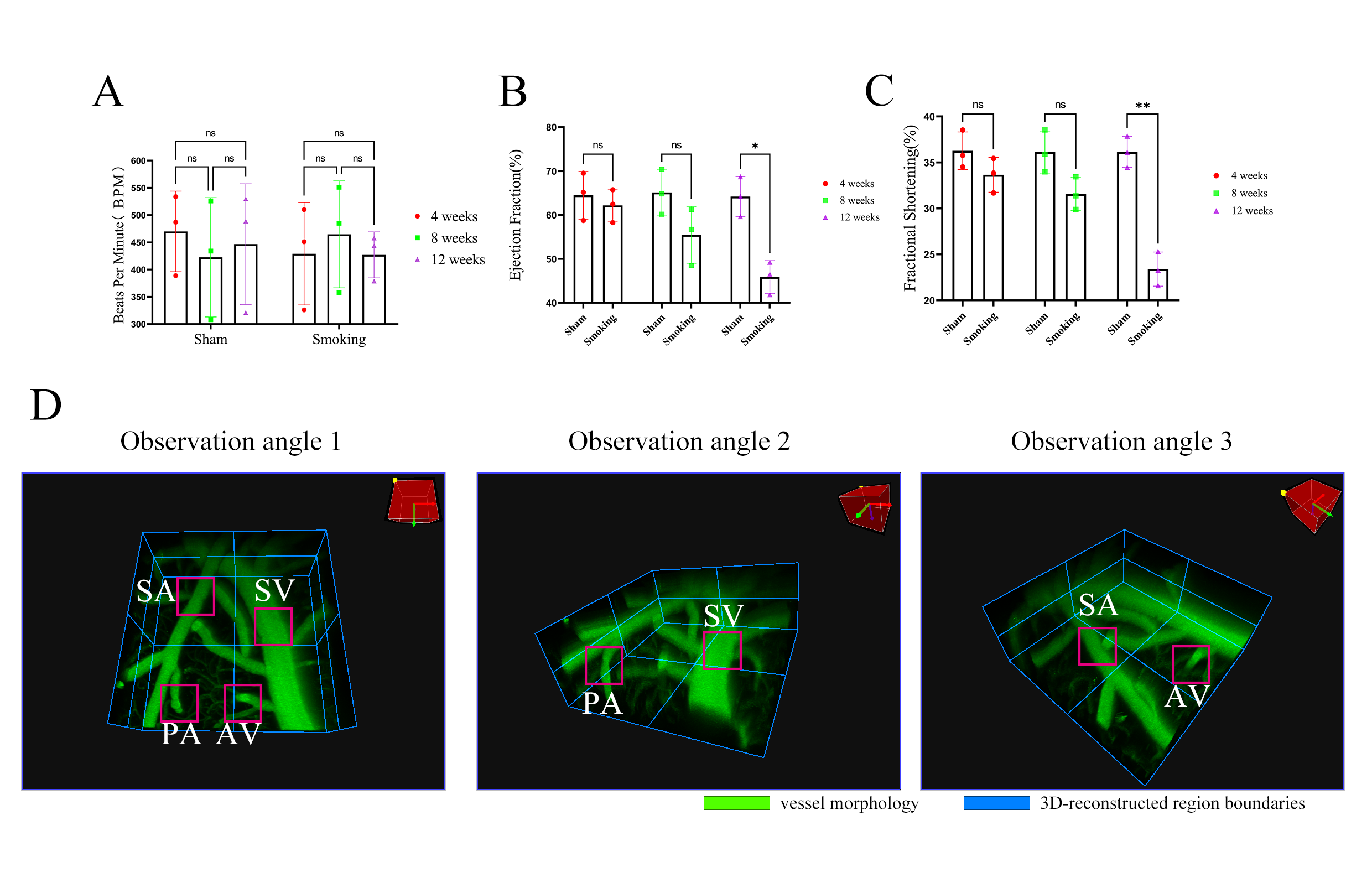

Supplement: Supplementary file 2 — Figure S2: Evaluation of cardiac function and 3D reconstruction of cerebrovascular morphology. (A) Quantification of beats per minute (BPM) in mice following chronic smoking exposure (n = 3 mice per group). Statistical analysis was performed using one‐way ANOVA. (B) Quantification of left ventricular ejection fraction (EF) in mice following chronic smoking exposure (n = 3 mice per group). Statistical analysis was performed using one‐way ANOVA. (C) Quantification of left ventricular fractional shortening (FS) in mice following chronic smoking exposure (n = 3 mice per group). Statistical analysis was performed using one‐way ANOVA. (D) Different angles of 3D reconstruction of vessel morphology showing the spatial relationship of surface arteries (SA), surface veins (SV), penetrating arteries (PA), and ascending veins (AV). Green indicates vessel morphology; blue lines indicate 3D‐reconstructed region boundaries. All data are shown as mean ± SD. *p < 0.05, **p < 0.01. AV, ascending veins; EF, ejection fraction; FS, fractional shortening; PA, penetrating arteries; SA, surface arteries; SD, standard deviation; SV, surface veins. [file CNS-32-e71040-s009.tif]

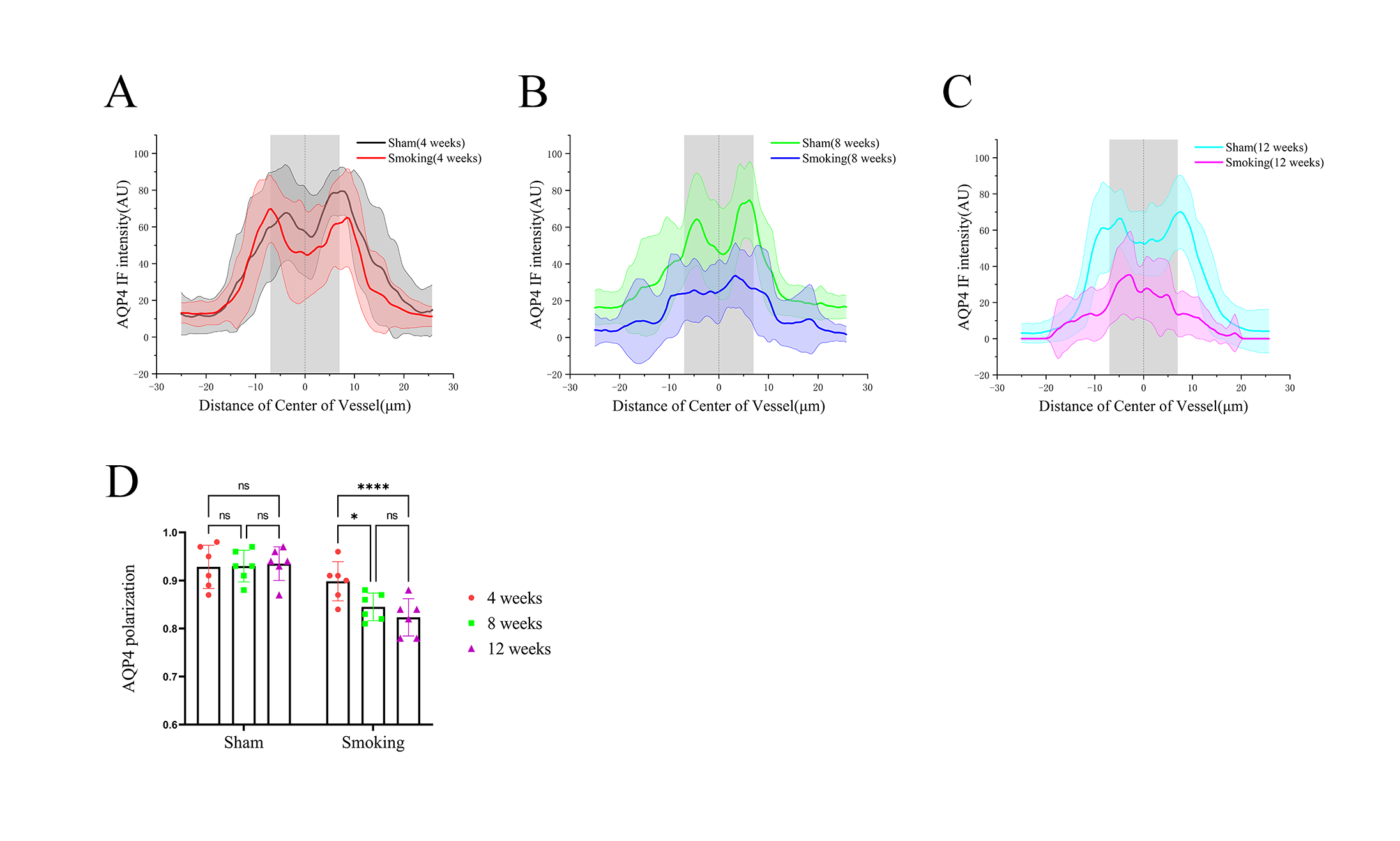

Supplement: Supplementary file 3 — Figure S3: Alterations in perivascular AQP4 distribution following chronic smoking. (A–C) Quantification of AQP4 immunofluorescence intensity profiles across large cortical vessels in the mouse brain following 4 weeks (A), 8 weeks (B), and 12 weeks (C) of exposure. The graph shows solid lines for mean values with standard error (SE) represented as shading. (D) Quantification of AQP4 polarization in the mouse brain following chronic smoking exposure. n = 6 mice per group. Statistical analysis was performed using one‐way ANOVA. Data in (D) are presented as mean ± SD. *p < 0.05, ****p < 0.0001. [file CNS-32-e71040-s007.tif]
